# Predictive Value of the Modified GRACE Scoring System for All-Cause Mortality in Patients with Acute Myocardial Infarction

**DOI:** 10.31083/j.rcm2406161

**Published:** 2023-06-06

**Authors:** Ju Yan, Chang-Jiang Deng, Si-Fan Wang, Mikereyi Aimaitijiang, Ting-Ting Wu, Ying-Ying Zheng, Xiang Xie, Yi-Tong Ma

**Affiliations:** ^1^Department of Cardiology, The First Affiliated Hospital of Xinjiang Medical University, 830011 Ürümqi, Xinjiang, China

**Keywords:** all-cause mortality (ACM), acute myocardial infarction (AMI), modified GRACE score (mGRACE), B-type natriuretic peptide precursor (BNP)

## Abstract

**Background:**

To establish a modified Global Registry of Acute Coronary Events (GRACE) scoring system with an improved predictive performance compared with the traditional GRACE scoring system.

**Methods:**

We identified 5512 patients who were hospitalized with a definite diagnosis of acute myocardial infarction (AMI) from January 1, 2015, to December 31, 2020, at the Heart Center of the First Affiliated Hospital of Xinjiang Medical University through the hospital’s electronic medical record system. A total of 4561 patients were enrolled after the inclusion and exclusion criteria were applied. The mean follow-up was 51.8 ± 23.4 months. The patients were divided into dead and alive groups by endpoint events. The differences between the two groups were compared using the two-sample *t* test and chi-square test. Adjusted traditional risk factors as well as LogBNP (B-type natriuretic peptide precursor, BNP) and the modified GRACE scoring system were included in a multifactorial COX regression model. The predictive performance of the traditional and modified GRACE scoring systems was compared by (Receiver Operating Characteristic) ROC curves.

**Results:**

Significant differences in age, heart rate, creatinine, uric acid, LogBNP, traditional GRACE score, and modified GRACE score were found between the dead and alive groups by the two-sample *t* test. Comparison of the two groups by the chi-square test revealed that the dead group had a higher incidence of males; higher cardiac function class; a previous history of hypertension, diabetes, coronary artery disease (CAD), or cerebrovascular disease; a history of smoking; the need for intra-aortic balloon pump (IABP) support; and more patients taking aspirin, clopidogrel, ticagrelor, and β-blockers. The results were analyzed by a multifactorial COX regression model, and after adjusting for confounders, age, cardiac function class, history of CAD, use of aspirin and β-blockers, and the modified GRACE scoring system were found to be associated with all-cause mortality (ACM) in patients with AMI. The ROC curve was used to compare the predictive performance of the conventional GRACE scoring system with that of the modified GRACE scoring system, and it was found that the modified GRACE scoring system (Area Under Curve (AUC) = 0.809, *p *< 0.001, 95% (Confidence Interval) CI (0.789–0.829)) was significantly better than the traditional GRACE scoring system (AUC = 0.786, *p *< 0.001, 95% CI (0.764–0.808)), the comparison between the two scores was statistically significant (*p *< 0.001). The change in the C statistic after 10-fold crossover internal validation of the modified GRACE score was not significant, and the integrated discrimination improvement (IDI) between the old and new models was calculated with IDI = 0.019 > 0, suggesting that the modified GRACE score has a positive improvement on the traditional GRACE score.

**Conclusions:**

The modified GRACE scoring system, established by combining B-type natriuretic peptide precursor (BNP) and the traditional GRACE scoring system, was independently associated with ACM in patients with AMI, with a larger AUC and higher predictive value than the traditional GRACE scoring system.

**Clinical Trial Registration:**

NCT02737956.

## 1. Introduction

In 1979, the World Health Organization, in an attempt to monitor trends in cardiovascular disease, established the monitoring trends and determinants in cardiovascular disease (MONICA) study. A total of 41 different national centers and 118 constituent units participated in the study, which monitored the incidence, risk factors, mortality, cardiovascular adverse events and treatment of coronary heart disease in 15 million people aged 25–64 over the previous 10 years [1]. An epidemiological study by the Framingham group found that between 1990 and 2010, global deaths from cardiovascular and circulatory diseases increased by 1/3 [2]. According to the 2021 study of the China Cardiovascular Health and Disease report [3], the prevalence rate of cardiovascular disease in China is continuously rising, and the mortality rate of cardiovascular disease is still one of the highest rates in China. Coronary heart disease is one of the main causes of cardiovascular deaths, and the death rate of acute myocardial infarction (AMI) due to coronary heart disease is on the rise. In 2013, the fifth health service survey in China showed that the prevalence rate of coronary heart disease in people over 15 years old was 10.2%, and the prevalence rate of coronary heart disease in people over 60 years old was 27.8%. It is anticipated that the number of patients suffering from AMI will also increase as the population continues to age. The risk of death in patients with AMI is also increasing. The China-PEACE study observed that although the absolute number of AMI patients receiving percutaneous coronary interventions (PCI) in China has significantly increased in the past 10 years, the hospital mortality and long-term prognosis of AMI patients have not significantly improved [4].

Both domestic and international studies have shown that cardiovascular disease has a high global burden of disease and mortality and that even with advances in treatment techniques and methods, there has been no significant improvement in in-hospital mortality or long-term survival. Therefore, a series of predictive scoring systems for the diagnosis of patients with coronary artery disease (CAD) and the prediction of major cardiovascular adverse events during hospitalization and in the long term were developed by Granger CB *et al*. [5], who combined age, heart rate, systolic blood pressure, serum creatinine, Killip classification, presence of cardiac arrest, presence of ST-segment bias, and presence of elevated cardiac enzymes to develop a predictive model for death during hospitalization in patients with acute coronary syndrome (ACS). This prediction model was later evaluated and used by Fox KAA *et al*. [6] for the prediction of death within 6 months in ACS patients, and has been termed the Global Registry of Acute Coronary Events (GRACE) scoring system. To date, this prediction scoring system has been used in major hospitals worldwide to predict in-hospital as well as 6-month mortality in ACS patients and can be used to stratify early intervention and treatment of high-risk patients.

With the continuous development of science and technology, biomarkers have emerged, and some new risk factors have been identified and used in clinical practice. Some studies have found that biomarkers such as serum B-type natriuretic peptide precursor (BNP) [7], calcitoninogen [8], cardiac troponin (cTn) [9, 10], highly sensitive C-reactive protein (Hs-CRP) [11], D-dimer [12], and Interleukin-6 (IL-6) [13] levels are associated with the occurrence of cardiovascular disease. Biomarkers such as creatine kinase MB (CK-MB), methemoglobin (MYO), cTnI and plasma N-terminal pro brain natriuretic peptide (NT-proBNP) levels are important for the early diagnosis of AMI. However, in a study on the correlation of NT-proBNP on in-hospital mortality in patients with acute ST-segment elevation myocardial infarction (STEMI) complicated by cardiogenic shock (CS), 64 patients with CS-STEMI were prospectively enrolled, and it was demonstrated that ROC analysis showed a strong relationship between elevated NT-proBNP and in-hospital mortality. Multiple regression analyses showed that NT-proBNP in STEMI patients was an independent predictor of death during hospitalization [14]. Additional studies confirmed that BNP is an independent predictor of death in AMI patients [7].

In the traditional GRACE scoring system, the primary population is older and includes fewer Asian or Chinese patient demographics and co-morbidities. Relevant cardiac markers and inflammatory indicators have been studied and found to be risk factors for adverse cardiovascular events in AMI patients; therefore, we combined the cardiac marker BNP with the traditional GRACE scoring system to establish a modified GRACE scoring system for in-hospital and long-term mortality in AMI patients in the Chinese or Xinjiang populations. The aim of this study is to identify and stratify patients early, thereby reducing in-hospital and long-term mortality in AMI patients.

## 2. Study Subjects and Methods

### 2.1 Study Subjects

A total of 5512 patients with AMI were identified from January 1, 2015, to December 31, 2020, in the First Affiliated Hospital of Xinjiang Medical University. The inclusion criteria were developed according to the fourth global definition of AMI in 2018. The details of the study design are registered at http://clinicaltrials.gov (NCT02737956).

#### 2.1.1 Inclusion Criteria

Inclusion criteria included troponin (cardiac troponin, cTn) dynamics with at least one value which exceeded the 99% reference limit and clinical evidence of at least one of the following acute myocardial ischemia criteria: (1) symptoms of acute myocardial ischemia; (2) new onset of ischemic electrocardiogram (ECG) changes; (3) formation of pathological Q waves; (4) imaging evidence of new onset of infarcted myocardium or localized ventricular wall motion abnormalities consistent with an ischemic etiology; and (5) coronary angiography, intracoronary imaging, or autopsy to identify coronary thrombus (not applicable to type 2 or 3 myocardial infarction) [15].

#### 2.1.2 Exclusion Criteria

**Fig. 1.**
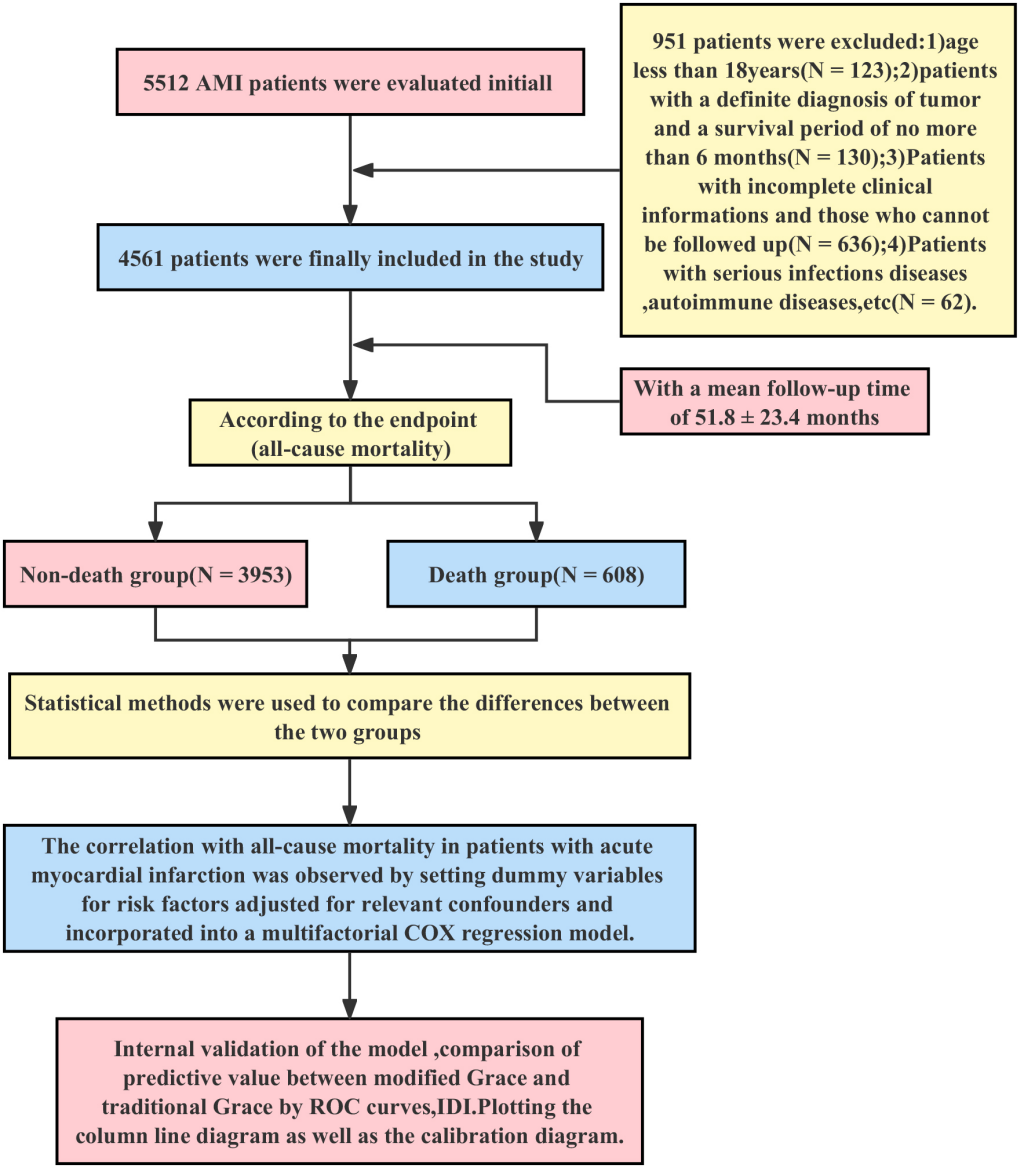
**Inclusion of research objects and flow chart**. AMI, acute myocardial infarction; GRACE, Global Registry of Acute Coronary Events; ROC, Receiver Operating Characteristic; IDI, integrated discrimination improvement.

The exclusion criteria for patients with AMI (including acute STEMI and acute non-ST-segment elevation myocardial infarction (N-STEMI)) were as follows: (1) age less than 18 years (N = 123); (2) patients with a definite diagnosis of tumor and a survival period of no more than 6 months (N = 130); 3 patients with incomplete clinical information and those who could not be followed (N = 636); (3) patients with serious infectious diseases and autoimmune diseases (N = 62). After inclusion and exclusion criteria were applied, 4561 patients with CAD were finally included in this study (Fig. 1).

### 2.2 Study Methods

#### 2.2.1 Collect Indicators

2.2.1.1 General Data

We collected patient demographic data using the hospital’s electronic medical record system. This data included age, sex, history of smoking and alcohol consumption, hypertension, diabetes, CAD, PCI, previous Coronary Artery Bypass Graft (CABG) surgery, cerebrovascular disease, hyperlipidemia, and vital signs such as heart rate and systolic blood pressure.

2.2.1.2 Clinical Data

The clinical data included creatinine, uric acid (UA), total cholesterol (TC), triglycerides (TG), high density lipoprotein cholesterol (HDL-C), low density lipoprotein cholesterin (LDL-C), creatine kinase, total bilirubin, total protein, homocysteine, ultrasensitive C-reactive protein (CRP), and B-type natriuretic peptide precursor (BNP) levels; cardiac function class (Killip class); cardiac arrest after admission; ST-segment changes on the ECG; use of aortic balloon counterpulsation during coronary angiography or stenting; need for thrombus aspiration; and the use of medications (aspirin, clopidogrel, ticagrelor, tirofiban, and beta-blockers).

#### 2.2.2 Diagnostic Criteria

The diagnostic criteria for hypertension were as follows: systolic blood pressure ≥140 mmHg, diastolic blood pressure ≥90 mmHg, and the need for blood pressure-lowering drugs within the last two weeks [16]. The diagnostic criteria for diabetes mellitus were as follows: fasting blood glucose ≥7.0 mmol/L or random blood glucose or 2-hour postprandial blood glucose ≥11.1 mmol/L, glycated hemoglobin ≥6.5%, or recent use of hypoglycemic drugs or insulin [17]. History of CAD included (1) percutaneous coronary intervention (PCI); (2) coronary artery bypass grafting (CABG); (3) inpatient diagnosis of myocardial infarction; (4) previous symptoms of chest pain; and (5) electrocardiogram and laboratory tests (cardiac enzymes, troponin). One of the following ancillary tests needs to be performed for confirmation: (1) electrocardiogram exercise testing; (2) coronary artery CT; (3) coronary angiography; and (4) echocardiography and myocardial nuclear angiography nuclear loading test. For stroke history, ischemic stroke diagnosis was based on symptoms/signs, mainly focal neurological deficits, with weakness or numbness of one side of the face or limb and speech impairment or full neurological deficits, and imaging with infarct lesions and CT/MRI to exclude cerebral hemorrhage [18].

### 2.3 Follow-up

Follow-up was performed mainly by telephone and hospital readmission, with a mean follow-up of 51.8 ± 23.4 months. Telephone follow-up was conducted after discharge to consult with patients and their families about any medication adjustments and endpoint events after discharge. Patients and their families were consulted about the reasons for hospitalization and the occurrence of endpoint events.

### 2.4 Endpoint

The follow-up endpoint event was all-cause mortality (ACM) during hospitalization and follow-up. ACM is a population study concept that refers to total deaths from all causes over a given period of time, which includes deaths from any cause during hospitalization and subsequent follow-up.

### 2.5 Statistical Methods

SPSS 21.0 (IBM, Armonk, NY, USA) and R 4.1.0 (https://cran.r-project.org/) statistical analysis software were used to analyze and process the data. The measurement data were first tested for normal distribution. ($\bar{x}\pm s$) was used for measurement data conforming or approximately conforming to normal distribution. Median and interquartile range (M, P25–P75) were used for nonnormal measurement data, and the number of cases (percentage) was used for counting data. Comparison of measurement data between two groups of ACM was performed by the two-sample *t* test, and comparison of counting data was performed by 2 test. BNP was log-transformed and incorporated into the original GRACE scoring system to establish the modified GRACE scoring system. The dummy variables (Q1, Q2, Q3, and Q4) were transformed for the modified GRACE scoring system. The dummy variables of the transformed modified GRACE scoring system were compared with related indicators, and the dummy variables of the above indicators were also transformed and compared. Multivariate COX regression models were used to clarify whether LogBNP, the modified GRACE score, and ACM were correlated. The log-rank test was used to compare the predictive performance between modified GRACE and traditional GRACE by constructing cumulative survival curves for endpoints using the Kaplan‒Meier method. *p *< 0.05 was considered to be significantly different.

### 2.6 Traditional GRACE Scoring System

The conventional GRACE scoring system includes the variables of the Killip classification, systolic blood pressure, heart rate, age, serum creatinine level, cardiac arrest, presence or absence of ST-segment bias, and presence or absence of elevated muscle enzymes [5, 6].

### 2.7 Data Quality Control

Prior to data collection, the content of the subject is determined, the collection index is clearly defined, the form for data collection is developed, and the content of the form for data collection is quality-controlled by the individual in charge of the patient’s data. The data collection staff and follow-up staff are specially trained by a specialized individual after the completion of the quality control. After the training process, the patient’s data is collected by two individuals. And if there is a difference, then a third individual and the primary designer of the study compare the data and perform quality control measures to determine what clinical and follow-up data should be included.

## 3. Results

The groups were divided into the dead and alive groups by the endpoint event of all-cause death at follow-up. The two groups were compared by the two-sample *t* test for age, heart rate, creatinine, UA, TG, TC, HDL-C, LDL-C, creatine kinase isoenzyme, LogBNP, conventional GRACE score, and modified GRACE score. In the comparison between the two groups, significant differences were found for age, heart rate, creatinine, uric acid, LogBNP, conventional GRACE score, and modified GRACE score (Table 1).

**Table 1.** **Comparison of quantifiable data between two groups**.

		Alive group	Dead group	χ ^2^	*p*
Sex [n (%)]	0	675 (17.1%)	163 (26.8%)	33.289	0.000
	1	3278 (82.9%)	445 (73.2%)		
Cardiac functional grading [n (%)]	I	249 (6.5%)	6 (1.1%)	605.429	0.000
	II	2389 (62.5%)	156 (27.6%)		
	III	903 (23.6%)	173 (30.6%)		
	IV	283 (7.4%)	230 (40.7%)		
Past medical history					
Hypertension [n (%)]	0	2101 (53.1%)	235 (38.7%)	44.332	0.000
	1	1852 (46.9%)	373 (61.3%)		
DM [n (%)]	0	2997 (75.8%)	404 (66.4%)	24.387	0.000
	1	956 (24.2%)	204 (33.6%)		
CHD [n (%)]	0	3312 (83.8%)	458 (75.3%)	26.282	0.000
	1	641 (16.2%)	150 (24.7%)		
PCI [n (%)]	0	3600 (91.1%)	540 (88.8%)	3.196	0.074
	1	353 (8.9%)	68 (11.2%)		
CABG [n (%)]	0	3932 (99.5%)	601 (601%)	3.321	0.068
	1	21 (0.5%)	7 (1.2%)		
Hyperlipidaemia [n (%)]	0	3931 (99.4%)	605 (99.5%)	0	1.000
	1	22 (0.6%)	3 (0.5%)		
Stroke [n (%)]	0	3767 (95.3%)	525 (86.3%)	75.987	0.000
	1	186 (4.7%)	83 (13.7%)		
Smoking [n (%)]	0	2277 (57.6%)	406 (66.8%)	18.312	0.000
	1	1676 (42.4%)	202 (33.2%)		
Drinking [n (%)]	0	3048 (77.1%)	489 (80.4%)	3.339	0.068
	1	905 (22.9%)	119 (19.6%)		
Use of apparatus					
Thrombus aspiration [n (%)]	0	3810 (96.4%)	592 (97.4%)	1.522	0.217
	1	143 (3.6%)	16 (2.6%)		
IABP [n (%)]	0	3864 (97.7%)	581 (95.6%)	10.190	0.001
	1	89 (2.3%)	27 (4.4%)		
Drug use					
Aspirin [n (%)]	0	453 (11.5%)	336 (55.3%)	706.740	0.000
	1	3500 (88.5%)	272 (44.7%)		
Clopidogrel [n (%)]	0	1591 (40.2%)	371 (61%)	92.754	0.000
	1	2362 (59.8%)	237 (39%)		
Ticagrelor [n (%)]	0	2858 (72.3%)	560 (92.1%)	110.066	0.000
	1	1095 (27.7%)	48 (7.9%)		
Tirofiban [n (%)]	0	3930 (99.4%)	607 (607%)	0.928	0.204
	1	22 (0.6%)	1 (0.2%)		
β-blocker [n (%)]	0	952 (24.1%)	383 (63%)	385.369	0.000
	1	3001 (75.9%)	225 (37%)		

DM, diabetes mellitus; CHD, coronary heart disease; PCI, Percutaneous Transluminal Coronary Intervention; CABG, Coronary Artery Bypass Grafting; IABP, intra-aortic balloon pump. *p *< 0.05 was statistically significant.

In the comparison between the two groups, it was found that there were more males; higher cardiac function class; a history of hypertension, diabetes, CAD, cerebrovascular disease; smoking; the need for an intra-aortic balloon pump (IABP); and a higher incidence of the use of aspirin, clopidogrel, ticagrelor, and β-blockers in the dead group than in the alive group (Table 2).

**Table 2.** **Comparison of measurement data between two groups**.

	Alive group	Dead group	*p*	T	95% CI	
					Lower	Upper
Age (years)	58.56 ± 12.30	68.70 ± 12.79	<0.001	–18.826	–11.195	–9.083
HR (times/minutes)	80.73 ± 16.00	87.63 ± 20.72	<0.001	–7.753	–8.640	–5.148
Scr (mmol/L)	85.03 ± 162.23	129.75 ± 260.06	<0.001	–4.087	–66.201	–23.234
UA (mmol/L)	340.61 ± 133.20	383.88 ± 158.47	<0.001	–6.350	–56.653	–29.895
TG (mmol/L)	1.89 ± 3.51	1.61 ± 1.38	0.065	1.848	–0.017	0.582
TC (mmol/L)	3.90 ± 1.18	3.83 ± 1.21	0.228	1.206	–0.041	0.172
HDL-C (mmol/L)	0.98 ± 2.09	1.20 ± 6.35	0.418	–0.810	–0.764	0.318
LDL-C (mmol/L)	3.55 ± 60.77	2.42 ± 0.96	0.431	0.666	–4.000	6.255
CK-MB (mmol/L)	77.02 ± 551.46	74.99 ± 303.49	0.936	0.081	–47.216	51.272
LogBNP	2.73 ± 0.71	3.32 ± 0.71	<0.001	–17.715	–0.659	–0.527
GRACE	148.97 ± 34.25	191.93 ± 43.16	<0.001	–23.433	–46.554	–39.357
mGRACE	–2.69 ± 1.21	–1.08 ± 1.36	<0.001	–25.633	–1.735	–1.488

HR, Heart rate; Scr, Serum creatinine; UA, Uric acid; TG, Triglyceride; TC, Total cholesterol; HDL-C, High density lipoprotein cholesterol; LDL-C, Low density lipoprotein cholesterin; CK-MB, Creatine kinase-MB; BNP, B-type natriuretic peptide precursor; GRACE, Global Registry of Acute Coronary Events. *p *< 0.05 was statistically significant.

The risk factors (age, sex, hypertension, diabetes, cardiac function class, CAD, previous PCI, hyperlipidemia, cerebrovascular disease, smoking, alcohol consumption, the need for an IABP, aspirin, clopidogrel, ticagrelor, tirofiban, β-blockers, UA, TG, TC) and the modified GRACE scoring system were included in a multifactorial COX regression model to observe their correlation with ACM. The results showed that age, cardiac function class, history of coronary heart disease, administration of aspirin and β-blockers, and the modified GRACE scoring system were correlated with ACM in patients with AMI (Table 3).

**Table 3.** **COX regression analysis of all-cause mortality in patients with AMI**.

	B	SE	Wald	*p*	Exp (β)	95% CI	
						Lower	Upper
Age	0.019	0.005	16.437	<0.001	1.020	1.010	1.029
Sex	0.148	0.123	1.458	0.227	1.160	0.912	1.475
Hypertension	0.163	0.103	2.531	0.112	1.177	0.963	1.440
DM	–0.043	0.107	0.165	0.685	0.958	0.777	1.180
Cardiac functional grading			22.783	<0.001			
Cardiac functional grading (1)	0.518	0.519	0.768	0.381	1.679	0.527	5.350
Cardiac functional grading (2)	0.967	0.600	2.600	0.107	2.630	0.812	8.519
Cardiac functional grading (3)	1.281	0.607	4.459	0.035	3.601	1.096	11.830
CHD	0.349	0.129	7.334	0.007	1.418	1.101	1.826
PCI	–0.179	0.181	0.984	0.321	0.836	0.587	1.191
Hyperlipidaemia	0.656	0.713	0.845	0.358	1.927	0.476	7.801
Stroke	0.209	0.143	2.148	0.143	1.233	0.932	1.630
Smoking	0.057	0.123	0.219	0.640	1.059	0.833	1.346
Drinking	–0.046	0.140	0.110	0.741	0.955	0.726	1.256
IABP	0.177	0.225	0.621	0.431	1.194	0.768	1.857
Aspirin	–0.475	0.185	6.604	0.010	0.622	0.433	0.893
Clopidogrel	–0.242	0.184	1.722	0.189	0.785	0.547	1.127
Ticagrelor	–0.462	0.249	3.453	0.063	0.630	0.387	1.026
Tirofiban	–0.364	1.005	0.131	0.717	0.695	0.097	4.987
β-blocker	–0.453	0.128	12.471	<0.001	0.636	0.494	0.817
UA	0.001	0.000	3.214	0.073	1.001	1.000	1.001
TG	0.005	0.014	0.113	0.736	1.005	0.978	1.032
TC	0.028	0.039	0.508	0.476	1.028	0.952	1.111
mGRCAE			32.655	<0.001			
mGRCAE (1)	0.945	0.310	9.320	0.002	2.574	1.403	4.722
mGRACE (2)	1.238	0.303	16.660	<0.001	3.449	1.903	6.251
mGRCAE (3)	1.715	0.321	28.539	<0.001	5.556	2.961	10.422

DM, diabetes mellitus; CHD, coronary heart disease; PCI, Percutaneous Transluminal Coronary Intervention; IABP, intra-aortic balloon pump; UA, Uric acid; TG, Triglyceride; TC, Total cholesterol; GRACE, Global Registry of Acute Coronary Events. *p *< 0.05 was statistically significant.

**Fig. 2.**
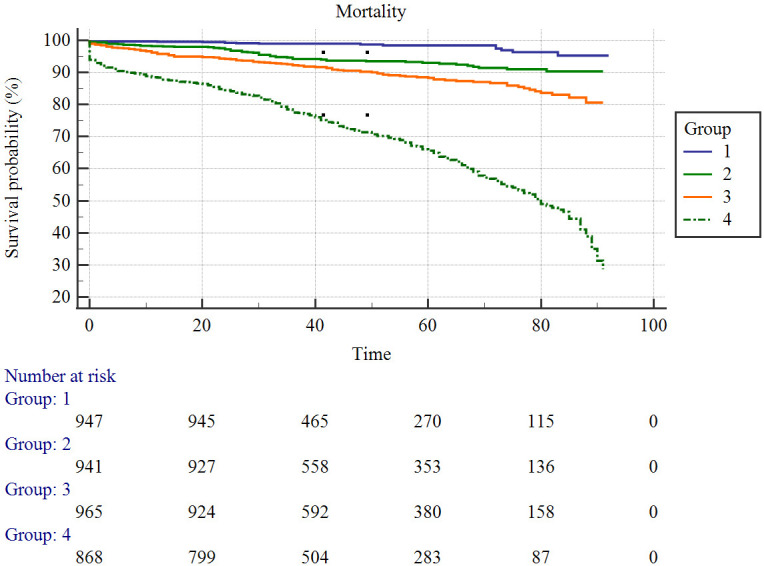
**Survival curves in patients with acute myocardial infarction**.

By subgroup analysis, 4561 patients with AMI were divided into four subgroups to observe the probability of all-cause death, and it was found that the survival rate was highest in the first subgroup and lowest in the fourth subgroup, and the comparison between the four subgroups was statistically significant (*p *< 0.001) (Fig. 2).

**Fig. 3.**
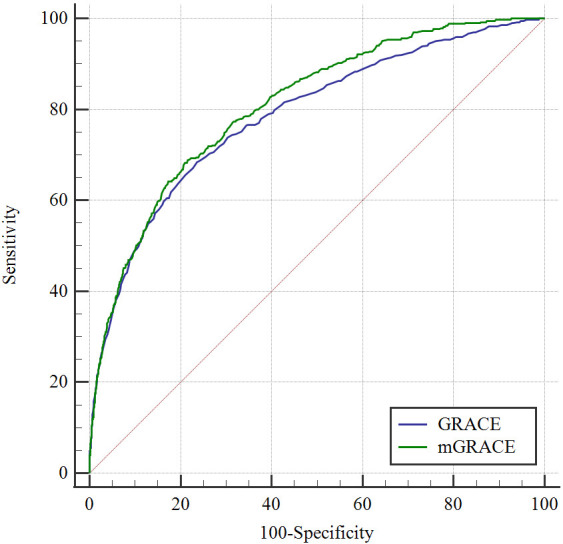
**Comparison of traditional GRACE score and modified GRACE score**. GRACE, Global Registry of Acute Coronary Events.

The ROC curve was used to compare the predictive performance of the conventional GRACE scoring system with that of the modified GRACE scoring system. The modified GRACE scoring system (AUC = 0.809, *p *< 0.001, 95% CI (0.789–0.829)) was better than the traditional GRACE scoring system (AUC = 0.786, *p *< 0.001, 95% CI (0.764–0.808)), the comparison between the two scores was statistically significant (*p *< 0.001) (Fig. 3).

**Fig. 4.**
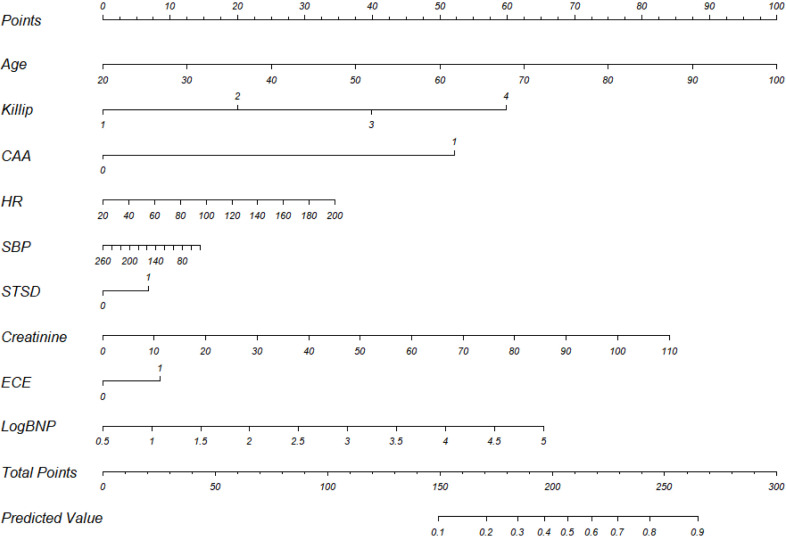
**Column line graph of modified GRACE scores**. CAA, Cardiac Arrest at Admission; HR, Heart Rate; SBP, systolic blood pressure; STSD, ST-Segment Deviation; ECE, Elevated Cardiac Enzyme Levels; BNP, B-type natriuretic peptide precursor; GRACE, Global Registry of Acute Coronary Events.

**Fig. 5.**
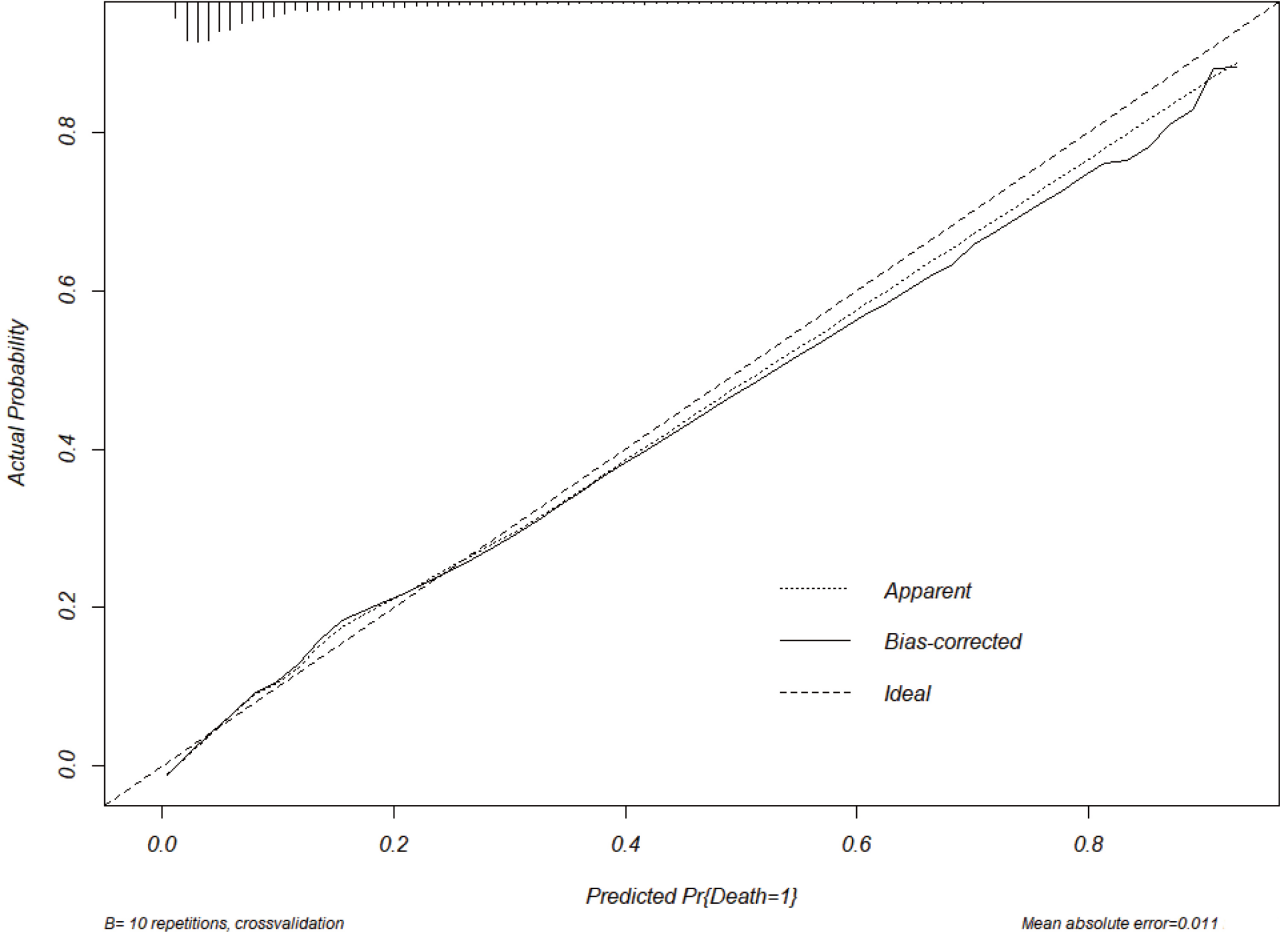
**Calibration chart for modified GRACE scoring system**. GRACE, Global Registry of Acute Coronary Events.

The study used K-fold cross-validation for internal validation. The original C-statistic was 0.821, and the C-statistic after 10-fold cross-validation was 0.817. The model was found to perform well based on the value of the C-statistic. The column line plot of the model for the modified GRACE score is shown in Fig. 4, and the calibration curve is shown in Fig. 5. The ROC curve was used to compare the traditional GRACE score and the modified GRACE score, and it was found that the area under the curve of the modified GRACE score was larger than that of the traditional GRACE score. Since the area under the curve of the two systems was not different, we calculated the integrated discrimination improvement (IDI), and the IDI = 0.019 > 0, suggesting that the modified GRACE score has a positive improvement over the traditional GRACE score.

## 4. Discussion

This study established a modified GRACE scoring system by modifying the traditional GRACE score by log-transforming the BNP level and combining it with the traditional GRACE score. The study participants were divided into a dead group and an alive group by the endpoint event of all-cause death at follow-up. A significant difference was found for age, heart rate, creatinine, UA, LogBNP, the traditional GRACE score, and the modified GRACE score between the two groups using the two-sample *t* test. Comparison of the two groups by the chi-square test revealed that males; a higher cardiac function class; patients with a previous history of hypertension, diabetes, CAD, and cerebrovascular disease; smoking; the need for an IABP; and the use of aspirin, clopidogrel, ticagrelor, and beta-blockers were more likely to be found in the dead group than in the alive group. Multifactorial COX regression models showed that LogBNP and the modified GRACE scoring system were associated with death in AMI patients, and the ROC curve revealed that the modified GRACE scoring system had a larger area under the curve than the conventional GRACE score, the change in the C statistic after 10-fold crossover internal validation of the modified GRACE score was not significant, and the integrated discrimination improvement (IDI) between the old and new models was calculated with IDI = 0.019 > 0, suggesting that the modified GRACE score has a positive improvement on the traditional GRACE score, which ultimately led to the conclusion that the modified GRACE scoring system had a higher predictive value and higher predictive performance than the conventional GRACE score.

A prediction model for death during hospitalization in ACS patients was established by Granger CB *et al*. [5], who combined age, heart rate, systolic blood pressure, serum creatinine, Killip classification, presence or absence of cardiac arrest, presence or absence of ST-segment bias, and presence or absence of elevated cardiac enzymes. This prediction model was later evaluated and used by Fox KAA *et al*. [6] in ACS patients within 6 months. However, the population included in the study was mainly European, and this study was conducted in an earlier period before the more widespread use of PCI for ACS patients. Since relevant cardiac markers were found to be risk factors for adverse cardiovascular events in AMI patients, we combined the cardiac marker BNP with the traditional GRACE scoring system to establish a modified GRACE scoring system. We developed an improved scoring system for AMI patients in the Chinese and Xinjiang populations to reduce in-hospital and long-term mortality.

A study by Sofidis G *et al*. [19] on the correlation between the GRACE score and the complexity of coronary artery lesions in ACS patients found that when classifying 539 patients with ACS according to the SYNTAX score, the GRACE score was a better predictor of severe CAD (SYNTAX ≥33). Our study reported that the GRACE score in ACS patients was significantly positively correlated with the SYNTAX score [19]. Related studies have also confirmed the significant value of the GRACE score in predicting the severity of coronary stenosis in ACS patients [20]. In an externally validated study of 300 patients with acute N-STEMI by Kumar D *et al*. [21], the GRACE risk score was a good predictor of in-hospital mortality in patients with NSTE-ACS. A retrospective cohort study by Baeza-Román A *et al*. [22] validated the accuracy of the GRACE score. In a subgroup analysis, they found that the GRACE score had good predictive value, good calibration and clinical applicability in the diabetes subgroup [22]. We found that the traditional GRACE score not only predicted in-hospital and out-of-hospital mortality in CAD patients but also correlated with the severity of coronary artery lesions. Previous studies confirmed the predictive reliability of the traditional GRACE score for the prognosis of CAD patients.

The traditional GRACE score has been found to correlate with coronary complications and death due to other cardiovascular medical conditions. It not only predicts the probability of MACE in the Takotsubo syndrome [23], but also predicts the probability of MACE events in patients with ACS combined with atrial fibrillation. It was found that both the GRACE and CHA2DS2-VASc scores predicted ACM, but GRACE was slightly more discriminative of ACM than CHA2DS2-VASc [24]. It can also be used to predict the risk of heart failure in ACS patients. Studies have found that each standard deviation increase in the GRACE score increases the risk of developing heart failure by more than twofold [25]. In other studies, STEMI patients with a moderate-to-high GRACE risk score who received fibrinolytic therapy followed by delayed coronary intervention had increased major cardiovascular events compared with patients with a low GRACE risk score [26]. The GRACE scores were also found to be associated with sex [27], age, degree of oxidative stress and inflammation [28], and nutritional status [29].

As models for predicting prognosis in patients with AMI have increased, some studies have compared models such as GRACE, HEART, ACEF, AGEF, TIMI and C-ACS. A study by Poldervaart JM *et al*. [30] comparing the GRACE, HEART, and TIMI scores in predicting the probability of major adverse cardiovascular events (MACE) in patients with chest pain in the emergency department found that the HEART score was superior to the GRACE and TIMI scores in differentiating patients with chest pain in terms of the occurrence of MACE when 1748 patients were scored and compared for their predictive performance. Another study confirmed that the predictive performance of the HEART score was higher than the TIMI and GRACE scores in predicting the probability of developing MACE in patients with chest pain [31]. However, in a study addressing the complexity of the GRACE, TIMI, and HEART scores on coronary vascular lesions in patients with ACS, it was found that the GRACE and HEART scores were positively correlated with predicting MACE in patients with non-ST-segment elevation ACS, but the TIMI scores were not. The combined use of the HEART and GRACE scores improves their accuracy for detecting coronary vascular complexity [32]. Further studies found that the AGEF risk score was superior to the GRACE, ACEF, and C-ACS risk scores in predicting in-hospital death in patients with ST-segment elevation ACS. In patients with non-ST-segment elevation ACS, the GRACE risk score was not significantly different from the AGEF risk score in predicting in-hospital mortality [33]. However, we found that each risk score system has its own characteristics, with better predictive performance in the medium term in patients with the characteristics involved in the scoring system, and the accuracy of its predictive performance in patients without its characteristic presentation needs to be confirmed by further studies.

However, with the continuous development of science and technology, biomarkers have been developed and new risk factors have been identified and used in clinical practice. Eggers KM *et al*. [34] and other researchers compared the value of different biomarkers on the prognosis of AMI patients and found different inflammatory features, coagulant activity, endothelial dysfunction, atherosclerosis, myocardial dysfunction and damage, apoptosis, renal function, glucolipid metabolism and 175 circulating biomarkers affecting the prognostic value of ACM, recurrent myocardial infarction, and heart failure hospitalization. This study found that BNP and GDF-15 (Growth-differentiation factor 15) have some value in the prognosis of AMI patients. Some new cardiac markers, such as TRAIL-R2 (Tumour necrosis factor-related apoptosis-inducing ligand receptor 2), CA-125 (carbohydrate antigen 125) and FGF23 (fibroblast growth factor 23), were also identified, but their clinical prognostic value needs to be confirmed in future studies [34].

Brain natriuretic peptide (BNP) was first isolated from porcine brain tissue as a cardiac natriuretic hormone, and later its gene was found on human chromosomes. Its secretion is mainly due to increased strain and mechanical load on the ventricular wall, which results in inhibition of the growth of cardiac as well as vascular cardiomyocytes, and ultimately leads to inhibition of the renin-angiotensin-aldosterone system which protects the myocardium from hypertrophy and fibrosis. In ACS patients, increased BNP concentrations are a predictor of myocardial infarction, heart failure and death, and can be used to assess the severity of ventricular function and heart failure [35, 36]. In a study on the correlation of NT-proBNP with in-hospital mortality in patients with acute STEMI complicated by cardiogenic shock involving 64 patients with CS-STEMI, ROC analysis showed a strong relationship between elevated NT-proBNP and in-hospital mortality. Multiple regression analysis showed that NT-proBNP was an independent predictor of death during hospitalization. A study by Gravning J *et al*. [37] on sensitive troponin assays and N-terminal B-type natriuretic peptide precursors (NT-proBNP) to predict coronary artery lesions and long-term prognosis in ACS found that NT-proBNP was superior to hs-cTnT and cTnI in predicting cardiovascular mortality by univariate and multivariate COX regression analysis at 1373 days of follow-up, and that NT-proBNP was associated with the presence of significant coronary artery lesions. The hs-cTn assay was superior to the standard cTnT assay in predicting significant coronary artery lesions in patients with NSTE-ACS, whereas NT-proBNP was superior to cTns in predicting long-term mortality [37]. Some studies have found that BNP can be used not only as a biomarker of poor prognosis following ACS but also as a drug for the treatment of AMI [38]. NT-proBNP concentrations are not invariable; in some patients with NSTEACS decreases in NT-proBNP concentrations are associated with chronic impairment of left ventricular function and increases in NT-proBNP concentrations are associated with acute myocardial injury [39]. In 2021, some investigators observed a poorer prognosis in nonobstructive coronary myocardial infarction and therefore modified the original GRACE score to create the GRACE 2.0 scoring system, which uses values derived from beta coefficients from regression models using nonlinear functions and subanalyses in cohorts defined by sex and type of MI. Their study found that in patients with MINOCA, the GRACE 2.0 score had a fairly high predictive accuracy for 1-year mortality [40]. Other studies of GRACE 2.0 in type 1 and type 2 myocardial infarction found that the GRACE 2.0 score provided good discrimination for all-cause death at 1 year in patients with type 1 myocardial infarction and moderate discrimination for those with type 2 myocardial infarction [41]. Sia CH *et al*. [42] found that although the traditional GRACE scoring system relies on a smaller population of Asian patients, in their study from Singapore, after establishing the SMIR risk score and comparing it to the GRACE 2.0 scoring system, the SMIR score performed as well as the GRACE 2.0 score in a multiethnic Asian AMI population undergoing PCI. A study by Fox KAA *et al*. [43] that modified the GRACE score in 32,037 patients with ACS found that using age, systolic blood pressure, pulse, and creatinine to create a GRACE risk score (2.0), the modified GRACE scoring system had better predictive performance as well as predictive value. Several other studies have found that the GRACE risk score, following adjustment for culprit coronary lesions undergoing PCI improves its predictive value for in-hospital mortality [44].

A study [45] on the prediction of cardiovascular events and death by cardiac risk scores and multiple biomarkers also found that BNP can be used as a prognostic indicator in AMI patients, and the combination of BNP with the conventional Grace score was found to have a higher predictive value. These results are similar to our own that both studies used the Grace score and myocardial markers to establish a modified Grace score. The results of both studies show that the combination of BNP and the traditional Grace score was found to have higher predictive value. The main differences are: firstly, the manuscript included a large number of study subjects (4561 AMI cases); secondly: the mean follow-up time of the study was 51.8 ± 23.4 months and the longest follow-up time was 91 months, based on which the study has some reliability. The results of these two studies are of great clinical importance for the prediction of ACM in patients with AMI; then, both studies were based on Grace score and myocardial markers to establish a modified Grace score, and the results of the studies showed that the combined BNP and traditional Grace score were found to have a higher predictive value, and they complemented and improved each other in terms of study population and follow-up time. The validation showed that the results of the study were highly reliable, and therefore we believe that both articles have profound clinical value and research significance.

The GRACE risk score system is an early risk scoring system used clinically to evaluate in-hospital mortality and long-term mortality in ACS patients and has good predictive value for coronary comorbidities and other diseases of the cardiovascular system. With the advent of biomarkers, basic and clinical studies have found a correlation between BNP and death in CAD patients. This has improved the traditional GRACE risk scoring system by combining the two systems to establish a new GRACE risk scoring system. Our study found that the modified GRACE risk scoring system has better predictive value than the traditional GRACE risk scoring system.

## 5. Conclusions

The modified GRACE scoring system, established by combining BNP and the traditional GRACE scoring system, was independently associated with ACM in patients with AMI, with a larger AUC and higher predictive value compared with the traditional GRACE scoring system.

## 6. Limitations

The present study is a single-center, large-sample retrospective cohort study, which will help to establish a prospective cohort study to further develop and validate the prediction model of AMI. In this study, we only included BNP cardiac markers. In future studies, we plan to collect other cardiac markers to further develop new predictive models for AMI. And there are still some indicators that are not collected, for example: time from chest pain onset to hospital arrival, door to balloon time to hospital, or PCI strategy (plain old balloon angioplasty, drug eluting stents or bare metal stents), etc. We will further collect relevant data at a later stage, supplement and improve the relevant contents of the database, and actively follow up to establish a predictive model with high clinical significance in order to guide clinical diagnosis and treatment.

## Data Availability

The datasets used and/or analyzed during the current study available from the corresponding author on reasonable request.
